# Effect of N- and C-Terminal Amino Acids on the Interfacial Binding Properties of Phospholipase D from *Vibrio parahaemolyticus*

**DOI:** 10.3390/ijms19082447

**Published:** 2018-08-19

**Authors:** Fanghua Wang, Ruixia Wei, Abdelkarim Abousalham, Wuchong Chen, Bo Yang, Yonghua Wang

**Affiliations:** 1 School of Food Science and Engineering, South China University of Technology, Guangzhou 510640, China; wangfanghua@scut.edu.cn (F.W.); fe1092917268@mail.scut.edu.cn (R.W.); wuchongexo@gmail.com (W.C.); 2 Institut de Chimie et de Biochimie Moléculaires et Supramoléculaires (ICBMS), Université Lyon 1, Univ Lyon, UMR 5246 CNRS, Métabolisme, Enzymes et Mécanismes Moléculaires (MEM2), Bât Raulin, 43 Bd du 11 Novembre 1918, CEDEX, F-69622 Villeurbanne, France; abdelkarim.abousalham@univ-lyon1.fr; 3 School of Bioscience and Bioengineering, South China University of Technology, Guangzhou 510006, China; yangbo@scut.edu.cn

**Keywords:** Phospholipase D, *Vibrio parahaemolyticus*, interfacial properties, monomolecular film, binding parameters

## Abstract

The effects of N-terminal (1–34 amino acids) and C-terminal (434–487 amino acids) amino acid sequences on the interfacial binding properties of Phospholipase D from *Vibrio parahaemolyticus* (VpPLD) were characterized by using monomolecular film technology. Online tools allowed the prediction of the secondary structure of the target N- and C-terminal VpPLD sequences. Various truncated forms of VpPLD with different N- or C-terminal deletions were designed, based on their secondary structure, and their membrane binding properties were examined. The analysis of the maximum insertion pressure (MIP) and synergy factor “*a*” indicated that the loop structure (1–25 amino acids) in the N-terminal segment of VpPLD had a positive effect on the binding of VpPLD to phospholipid monolayers, especially to 1,2-dimyristoyl-*sn*-glycero-3-phosphoserine and 1,2-dimyristoyl-*sn*-glycero-3-phosphocholine. The deletion affecting the N-terminus loop structure caused a significant decrease of the MIP and synergy factor *a* of the protein for these phospholipid monolayers. Conversely, the deletion of the helix structure (26–34 amino acids) basically had no influence on the binding of VpPLD to phospholipid monolayers. The deletion of the C-terminal amino acids 434–487 did not significantly change the binding selectivity of VpPLD for the various phospholipid monolayer tested here. However, a significant increase of the MIP value for all the phospholipid monolayers strongly indicated that the three-strand segment (434–469 amino acids) had a great negative effect on the interfacial binding to these phospholipid monolayers. The deletion of this peptide caused a significantly greater insertion of the protein into the phospholipid monolayers examined. The present study provides detailed information on the effect of the N- and C-terminal segments of VpPLD on the interfacial binding properties of the enzyme and improves our understanding of the interactions between this enzyme and cell membranes.

## 1. Introduction

Biological cell membranes are usually composed of phospholipid bilayers. It is well known that more than 25% of proteins bind to cell membranes and their function is highly dependent on the membrane–protein interactions [1,2,3]. Thus, constructing model membranes with less complicated lipid mixtures or individual lipids is very important to gain information on the binding of proteins to membranes [4,5]. Phospholipid monolayers are the most important and popular model among the various model membrane systems developed. The advantage of this model is that several physical parameters, such as the surface pressure (Π) of lipids, subphase content, and lipid composition, can be controlled precisely [6,7,8,9]. In addition, it has been proved that there is a direct thermodynamic relationship between bilayers and monolayers [10,11]. More importantly, only phospholipid monolayers allow to determine the affinity of compounds for the inner or the outer leaflet of the membrane bilayer separately [12]. Thus far, different interfacial absorption parameters have been employed to investigate how proteins interact with membranes. Phillips and Sparks (1980) first introduced the maximum insertion pressure (MIP) parameter to characterize protein absorption and lipid specificity [13]. The synergy factor “*a*” and the Δ*Π*_0_ (corresponding to the surface pressure increase from an initial surface pressure of 0 mN m^−1^) were further introduced to characterize interfacial binding properties [14]. The MIP value of proteins for lipid monolayers has been widely used to characterize protein adsorption and lipid specificity [15]. The synergy factor “*a*” has allowed to highlight the specificity of proteins for different phospholipids [14]. In addition, Bénarouche and colleagues (2013) reported a simple model to calculate the main kinetic constants, namely, the adsorption (*k_a_*) and the desorption (*k_d_*) kinetic constants and the enzyme–lipid interfacial adsorption equilibrium coefficient (*K_Ads_*) of lipase adsorption to phospholipid monolayers [16]. On the basis of these parameters (MIP, Synergy factor “*a*”, Δ*Π_0_*, *k_a_*, *k_d_*, *K_Ads_*), lipid–protein interactions and the membrane affinity of proteins for phospholipid monolayers could be explored in detail.

Phospholipase D (PLD; EC 3.1.4.4.) is a key lipolytic enzyme that catalyzes the hydrolysis of the distal phosphodiester bond of glycerophospholipids, generating phosphatidic acid (PA) and a free polar head group. In addition to the hydrolysis reaction, in the presence of primary alcohol, PLD could also catalyzes a transphosphatidylation reaction to form the corresponding phosphatidyl alcohol. By using these two reactions, PLDs have various functions in membrane degradation and reorganization, cell regulation, and even signal transduction [17,18]. As generally typical of lipid-converting enzymes, PLDs need to bound to the phospholipid interface to gain access to their substrates. Moreover, interfaces are very important for the full activity of PLDs [19]. However, despite the increasing number of PLDs identified in different organisms at the genetic level and the numerous reports on PLD-mediated synthesis of tailor-made phospholipids with functional head groups [20], information on the enzyme–membrane interaction is still largely lacking.

It has been found that the C-terminal and N-terminal domains of lipases affect protein adsorption to the membrane. For instance, Chahinian et al. (2002) proved that the C-terminal domain of human pancreatic lipase plays a critical role in the interfacial binding of the lipase [21]. Bussières et al. (2012) proposed the N- and C-terminal segments of lecithin:retinol acyltransferase allow to anchor this protein to the lipid bilayer [22]. Sayari et al. (2005) concluded that the N-terminal peptide of *Rhizopus oryzae* (ROL32) lipase can significantly affect the specific activity, regioselectivity, and stereoselectivity of the lipase as well as the binding to its substrate [23]. In the present study, PLD from *Vibrio parahaemolyticus* (VpPLD) was successfully expressed in *Escherichia coli*. The aim was to gather information on the influence of the C- and N-terminal segments on the interfacial binding properties of PLD, to identify preferential binding features for various phospholipid monolayers. Thus, truncated mutants with different deletions of the N- and C-terminal segments were constructed, and their interfacial binding properties were compared with those of wild-type VpPLD. The present study is the first to provide detailed information on the function of the N- and C-terminal segments in the interfacial binding of VpPLD and improves our understanding of how this enzyme interacts with cell membranes.

## 2. Results and Discussion

### 2.1. Analysis of the Primary and Secondary Structures of VpPLD-WT and Its N- and C-Terminal Sequences

**Figure 1:**
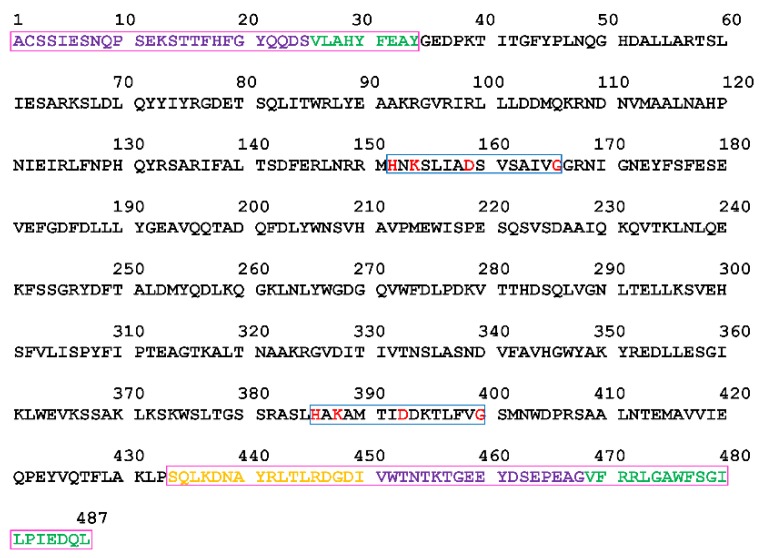
Full-length amino acid sequence of the mature Phospholipase D (PLD) from *Vibrio parahaemolyticus* (VpPLD). The two conserved HKD motifs are labeled by blue frames, and key residues within them are labeled in red. The N- and C-terminal peptides investigated in the present study are labeled by purple frames. Various amino acid sequences within these peptides are labeled with different colors.

The full-length sequence of VpPLD from *Vibrio parahaemolyticus* has been deposited in the NCBI-Protein databases under the accession number of EXJ48329.1. The full-length sequence of VpPLD is composed of 505 amino acids (aa). By using SignalP 4.1 server, the first 18 amino acids (MLHTLSKFIFAFMFSVLS) were predicted to be a signal peptide and indicated that the enzyme is an extracellular protein. The molecular mass and isoelectric point of the deduced mature VpPLD protein (VpPLD-WT) were predicted to be 55,239.06 Da and 5.35, respectively, by using the Compute pI/Mw tool at the ExPASy molecular biology web server of the Swiss Institute of Bioinformatics. Two highly conserved sequence motifs (HxK(x)_4_D(x)_6_G) comprising the active site residues were found in the VpPLD-WT enzyme, with histidine 152 and histidine 386 as the putative catalytic residues (Figure 1). The highly conserved motifs present in the primary structure provided a strong evidence that this protein is a member of the PLD superfamily.

**Figure 2:**
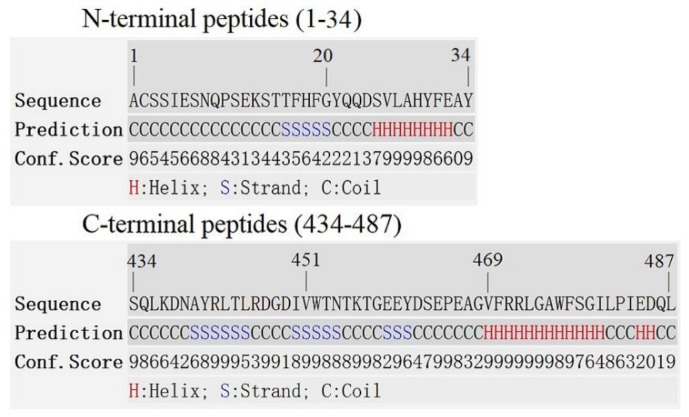
Predicted secondary structures of the N- and C-terminal amino acid sequences of PLD investigated in the present study, obtained using the I-TASSER server. To ensure the accuracy of the results, each sequence was analyzed using two additional online tools: Proteinprediction and SSPro. The three types of analysis yielded similar results. The secondary structures here presented are those with the highest confidence scores (Conf.Score). The confidence scores range from 0 to 9, which correspond, respectively, to the lowest and the highest confidence levels. H, Helix; S, strand or β-sheet; C, coil.

In the present study, the effect of N- and C-terminal amino acids on the interfacial binding of VpPLD to different phospholipid monolayers was investigated. For the N-terminal segment, the first 34 amino acids were considered. The primary structure of the first 34 amino acids was analyzed using different online tools to predict their secondary structure, which yielded very similar data (Figure 2). The N-terminal first 34-amino acid peptide was thus predicted to form a coil (aa 1–24) and a helix (aa 25–34) structure (Figure 2). Considering its amino acid composition (Table 1), the hydrophobic proportion of amino acids in the coil segment reaches 24%, and the hydrophilic proportion of amino acids (polar and charged) reaches 76%. This indicates that this segment is highly hydrophilic. Near the coil segment, a classical helical secondary structure was found (aa 25–34). This structure is composed of polar (22%) and charged (22%) amino acids, while hydrophobic amino acids in the helix reach 56% (Table 1). On the basis of these features, two truncation mutants were designed, one lacking the first 25 amino acids (VpPLD-(His)_6_-Δ1-25), and the other lacking the entire sequence of 34 amino acids (VpPLD-(His)_6_-Δ1-34). These deletions mutants were then employed to compare the functions of the helix and the coil segments in the interfacial binding of VpPLD to different phospholipid monolayers.

For the C-terminal peptides, the amino acids 434–487 were taken in consideration. The results from the secondary structure analysis indicated that this segment was mainly composed of three β-sheets (aa 434–468) and a helix (aa 469–487) (Figure 2). Prediction analysis of the three-dimensional structure of these peptides indicated that the three β-sheets compact, forming a stable structure. Considering the amino acid composition of the different portions of the segment, the helix (aa 469–487) is mainly composed of hydrophobic amino acids (68%), while for the β-sheet compact section (aa 434–468), the hydrophobic amino acids represent 34%, and the polar and charged hydrophilic amino acids are 11% and 34%, respectively Table 1). To investigate the function of the β-sheet compact section and the helix in the interfacial binding properties of the enzyme, three truncations were designed: the helix deletion ((His)_6_-VpPLD-Δ469-487), the complete deletion (helix and strands, (His)_6_-VpPLD-Δ434-487), and a deletion involving the three-β-sheet structure (His)_6_-VpPLD-Δ451-487). 

### 2.2. Mutants Construction and Purification

**Figure 3:**
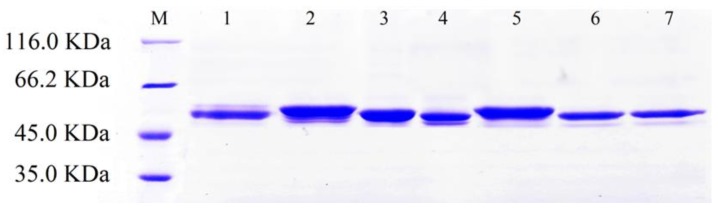
Sodium dodecyl sulphate-polyacrylamide gel electrophoresis (SDS-PAGE) analysis of the purified wild-type VpPLD and its corresponding mutants. Lane M, molecular mass marker (kDa); lane 1, purified (His)_6_-VpPLD-WT; lane 2, purified (His)_6_-VpPLD-Δ469-487; lane 3, purified (His)_6_-VpPLD-Δ451-487; lane 4, purified (His)_6_-VpPLD-Δ434-487; lane 5, purified VpPLD-(His)_6_-WT; lane 6, purified VpPLD-(His)_6_-Δ1-25; lane 7, purified VpPLD-(His)_6_-Δ1-34.

Since PLD adsorption at the lipid–water interface must occur before an insoluble substrate is hydrolyzed, the corresponding His152Ala mutant based on wild-type and various mutants was further constructed for studying such lipid-protein interactions independently from substrate hydrolysis. Moreover, to avoid the influence of the (His)_6_ tag on the binding of the protein to the monolayer, we constructed the N-terminus deletion mutants using the plasmid pET21a-VpPLD-WT as a template, which codes for a protein with the (His)_6_ tag at the C-terminus. Similarly, for the C-terminal truncation mutants, we used the plasmid pET28a-VpPLD-WT as a template, producing a protein with the (His)_6_ tag connected to the N-terminus. By using this protocol, the recombinant proteins could be purified by using Ni^2+^-nitrilotriacetic acid (NTA) Sepharose fast-flow columns. Selected transformant colonies were cultured in a shaking flask to measure protein expression. Proteins with high purity were obtained from the culture supernatants after two purification steps. The highly purified VpPLD-(His)_6_-WT, (His)_6_-VpPLD-WT, and teir truncated mutants were subjected to SDS-PAGE analysis. Single bands of approximately 55 kDa were detected in each sample (Figure 3). This molecular mass coincides with the predicted molecular weight of 55,239.06 Da and confirmed the purification of the desired proteins.

### 2.3. Binding of VpPLD-WT and Its N- and C-Terminal Truncated Mutants to an Air–Water Interface

**Figure 4:**
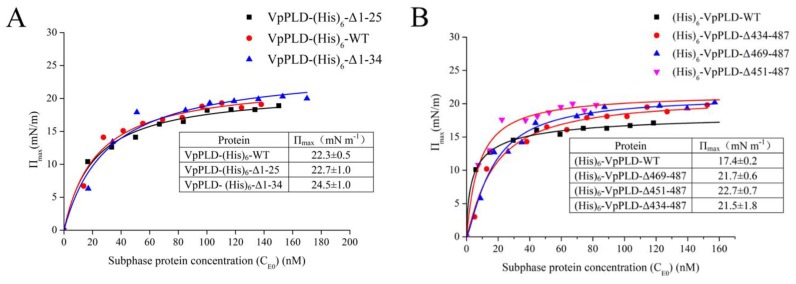
Increase of the surface pressure as a function of subphase protein concentration (C_E0_) in the absence of a phospholipid monolayer. (**A**) Wild-type VpPLD (VpPLD-(His)_6_-WT) and its N-terminal truncated mutants. (**B**) Wild-type VpPLD ((His)_6_-VpPLD-WT) and its C-terminal truncated mutants. Wild-type VpPLD and its corresponding N- and C-terminal truncated mutant proteins at different concentrations were injected beneath an air–water interface, and the surface pressure increase was recorded until equilibrium. The maximum surface pressure increase (Π_max_) was then obtained for each sample. Buffer used for the sub-phase: 50 mM Tris-HCl, pH 8.0. For the detailed method, please see Section 3.5.

We measured the ΔΠ necessary for the surface pressure (Π_e_) to reach equilibrium using several different protein concentrations in the absence of a lipid monolayer. We thus obtained the maximum surface pressure increase (Π_max_) for each protein concentration. As can be seen in Figure 4A, the Π_max_ for VpPLD-(His)_6_-WT, VpPLD-(His)_6_-Δ1-25, and VpPLD-(His)_6_-Δ1-34 were 22.3 ± 0.5, 22.7 ± 1.0, and 24.5 ± 1.0 mN m^−1^, respectively. No significant difference was found between these three groups. By contrast, for the C-terminal truncation, the Π_max_ for (His)_6_-VpPLD-WT was 17.4 ± 0.2 mN m^−1^, while the Π_max_ for (His)_6_-VpPLD-Δ469-487, (His)_6_-VpPLD-Δ451-487, and (His)_6_-VpPLD-Δ434-487 increased to 21.67 ± 0.6, 22.7 ± 0.7, and 21.5 ± 1.8 mN m^−1^, respectively (Figure 4B). These results indicated that the C-terminal deletion significantly improved the binding properties of VpPLD to the air–water interface. Moreover, a significant difference was also found between VpPLD-(His)_6_-WT (22.3 ± 0.5 mN m^−1^) and (His)_6_-VpPLD-WT (17.4 ± 0.2 mN m^−1^) (*p* < 0.05). This results strongly indicate that the position of the (His)_6_ tag has a strong influence on the binding of the protein to the air–water interface.

### 2.4. Binding of VpPLD-(His)_6_-WT and Its N-Terminal Truncated Mutants to Different Phospholipid Monolayers

**Figure 5:**
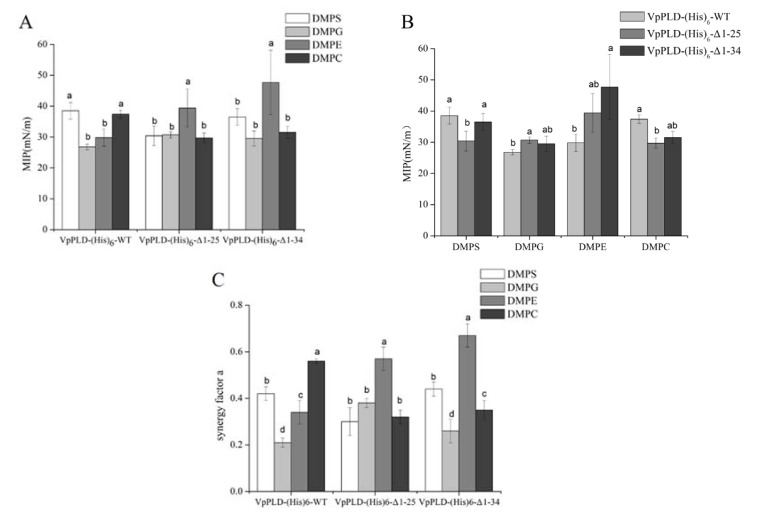
Maximum insertion pressure (MIP) (**A**,**B**) and Synergy factor *a* (**C**) of VpPLD-(His)_6_-WT and its N-terminal truncated mutants (VpPLD-(His)_6_-Δ1-25 and VpPLD-(His)_6_-Δ1-34) obtained in the presence of different phospholipid monolayers. The statistical analysis of the data allowed to determine the values which were significantly different, as indicated by the different letters (*p* = 0.05).

The extent of the binding of VpPLD-(His)_6_-WT and its N-terminal truncated mutants to different phospholipids (1,2-Dimyristoyl-*sn*-glycero-3-phosphocholine (DMPC), 1,2-Dimyristoyl-*sn*-glycero-3-phosphoethanolamine (DMPE), 1,2-Dimyristoyl-*sn*-glycero-3-phospho-*rac*-glyceol (DMPG), 1,2-Dimyristoyl-*sn*-glycero-3-phospho-l-serine (DMPS)) was performed at different Π_i_ of phospholipid monolayers. The MIP corresponds to the maximum surface pressure of a monolayer at which a peptide or protein can insert into the monolayer [12]. As shown in Figure 5A, for the VpPLD-(His)_6_-WT, the highest MIP values were found for DMPS (38.5 ± 2.7 mN m^−1^) and DMPC (37.4 ± 1.3 mN m^−1^). A statistical analysis indicated that there was no significant difference between these two groups. Similar MIP values were also found for DMPG (26.8 ± 0.9 mN m^−1^) and DMPE (29.8 ± 2.8 mN m^−1^), but they were significantly lower than those for DMPS and DMPC (*p* < 0.05). These results indicated that VpPLD-(His)_6_-WT binds preferentially to DMPS and DMPC monolayers. However, when analyzing VpPLD-(His)_6_-Δ1-25 and VpPLD-(His)_6_-Δ1-34, the highest MIP values were found for DMPE, corresponding to 39.4 ± 6.2 mN m^−1^ and 47.8 ± 8.7 mN m^−1^, respectively. Moreover, no significant difference was found between VpPLD-(His)_6_-Δ1-25 and VpPLD-(His)_6_-Δ1-34 for DMPE (*p* < 0.05). These results strongly indicated that the deletion of the first 25 amino acids of the N-terminu significantly changed the binding preference of VpPLD-(His)_6_-WT from DMPS/DMPC to DMPE.

Comparing the binding of each mutant to the different phospholipids, differences were also found that depended on the phospholipid monolayers. As can be seen from Figure 5B, for DMPS, the MIP value of VpPLD-(His)_6_-Δ1-25 (30.4 ± 3.2 mN m^−1^) was significantly lower than those of VpPLD-(His)_6_-WT (38.5 ± 2.7 mN m^−1^) and VpPLD-(His)_6_-Δ1-34 (36.5 ± 2.7 mN m^−1^). However, no significant difference was found between VpPLD-(His)_6_-WT (38.5 ± 2.7 mN m^−1^) and VpPLD-(His)_6_-Δ1-34 (36.5 ± 2.7 mN m^−1^) (*p* > 0.05). The same trends were also found for DMPC. We conclude that the presence of the first 25 amino acids at the N-terminal of VpPLD greatly enhances the binding of VpPLD to the DMPS and DMPC.

For DMPG, the MIP for VpPLD-(His)_6_-Δ1-25 and VpPLD-(His)_6_-Δ1-34 increased to 30.7 ± 1.0 mN m^−1^ (*p* < 0.05) and 29.5 ± 2.4 mN m^−1^ (*p* > 0.05), respectively, compared with VpPLD-(His)_6_-WT (26.8 ± 0.9 mN m^−1^) (Figure 5B). The statistical analysis indicated that this change was not significant. These results indicated that the deletion of amino acids 1–25 facilitated the binding of VpPLD to DMPG. A further deletion of amino acids 26–34 caused no significant change of the binding affinity, which means that the amino acids 26–34 have no effect on the binding to DMPG.

For DMPE, even though the MIP value for VpPLD-(His)_6_-Δ1-25 was higher than that of VpPLD-(His)_6_-WT, a significant change was only found between VpPLD-(His)_6_-Δ1-34 and VpPLD-(His)_6_-WT (*p* < 0.05), while no significant change was found between VpPLD-(His)_6_-Δ1-25 and VpPLD-(His)_6_-Δ1-34 (*p* > 0.05) (Figure 5B). From these results, we can conclude that the above observed change of the binding preference for VpPLD-(His)_6_-Δ1-25 was mainly caused by a decrease of the binding affinity of VpPLD for DMPS and DMPC, and not by an increase on the binding affinity for DMPE.

Synergy factor *a* is another parameter commonly employed to evaluate the binding properties of peptides and proteins to a monolayer. This parameter is obtained by increasing of one unit the slope of the plot of the surface pressure increase (ΔΠ) as a function of the initial surface pressure (Π_i_) [14]. It was shown that a positive synergy indicates a favorable binding of proteins. The MIP, in this case, corresponds to an insertion surface pressure. In the present study, all the proteins investigated had synergy factor *a* > 0 (Figure 5C), indicating that a positive interaction occurred between proteins and phospholipid monolayers.

It was nevertheless interesting to compare the MIP and synergy factor values. As mentioned above, no significant difference could be seen between the MIP of VpPLD-(His)_6_-Δ1-25 and that of VpPLD-(His)_6_-Δ1-34 in the presence of DMPE (Figure 5B), which was clearly supported by the synergy data (Figure 5C) (*p* < 0.05), with synergy values of 0.57 ± 0.05 and 0.67 ± 0.05, respectively. The small difference between the MIP of VpPLD-(His)_6_-WT and that of VpPLD-(His)_6_-Δ1-34 in the presence of DMPS, DMPG, and DMPC (Figure 5B) was also convincingly supported by the synergy data (*p* > 0.05) (Figure 5C). Similarly, the significant change between the MIP of VpPLD-(His)_6_-WT and that of VpPLD-(His)_6_-Δ1-25 in the presence of DMPS, DMPG, and DMPC (Figure 5B) was convincingly supported by the synergy data *a* (*p* < 0.05) (Figure 5C).

The results of MIP and of the synergy *a* analysis indicated that the first 1–25 amino acids in the N-terminal segments of VpPLD play an important role in VpPLD membrane binding, especially for DMPS and DMPC. In contrast, the helix structure (aa 26–34) basically has no influence on the binding of VpPLD to the phospholipid monolayer. These results are supported by the secondary structure and amino acid composition of the 1–25-aa peptide. Our above findings document that the secondary structure of the 1–25-amino acid segment is a classical loop, and, more importantly, the highly hydrophilic character (76%) of this segment facilitates the formation of electrostatic interactions with phospholipid monolayers of DMPS and DMPC. Removing these peptides may decrease the interaction of the protein with the monolayer and thus decrease its binding properties. 

### 2.5. Extent of the Binding of (His)_6_-WT and Its C-Terminal Truncated Mutants to Different Phospholipid Monolayers

**Figure 6:**
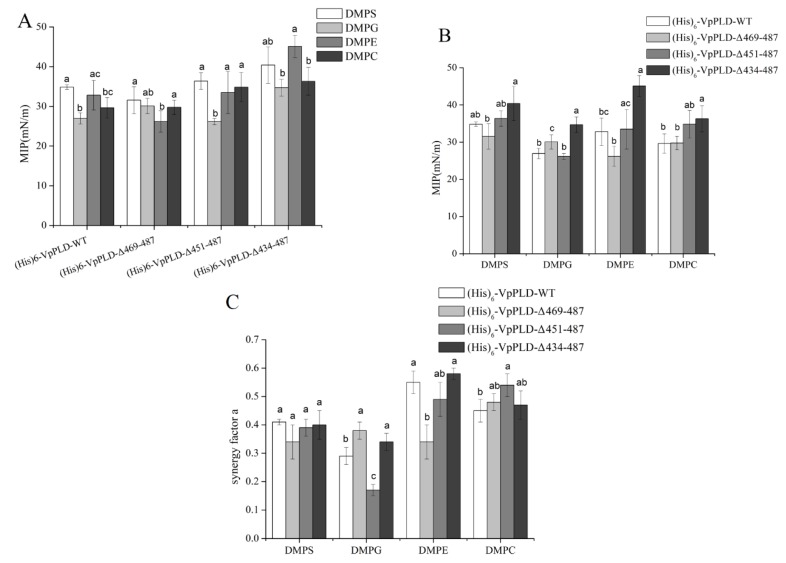
MIP (**A**,**B**) and Synergy factor *a* (**C**) of (His)_6_-VpPLD-WT and its C-terminal truncated mutants ((His)_6_-VpPLD-Δ469-487, (His)_6_-VpPLD-Δ451-487, and (His)_6_-VpPLD-Δ434-487) in the presence of different phospholipid monolayers. The statistical analysis of the data allowed to determine the values which were significantly different (labeled with different letters) (*p* = 0.05).

As can be seen in Figure 6A by comparing (His)_6_-VpPLD-WT, (His)_6_-VpPLD-Δ469-487, and (His)_6_-VpPLD-Δ451-487, the highest MIP value was found for DMPS. For (His)_6_-VpPLD-Δ434-487, the highest value was found for DMPE (45.1 ± 2.8 mN m^−1^). However, the statistical analysis indicated that, for (His)_6_-VpPLD-Δ434-487, there was no significant difference between the MIP values for DMPS (40.4 ± 4.6 mN m^−1^) and DMPE (45.1 ± 2.8 mN m^−1^) (*p* > 0.05). These results strongly indicate that the deletion the C-terminal peptide has no significant effect on the binding selectivity of VpPLD for the different phospholipid monolayers tested here.

Basically, no significant difference could be found between (His)_6_-VpPLD-WT, (His)_6_-VpPLD-Δ469-487, and (His)_6_-VpPLD-Δ451-487 for DMPS, DMPE, and DMPC. For example, for the DMPS, the MIP value for these three proteins was 34.8 ± 0.6 mN m^−1^, 31.2 ± 3.4 mN m^−1^, and 36.4 ± 2.1 mN m^−1^, respectively (*p* > 0.05). For DMPE, the MIP value for these three proteins was 32.8 ± 3.7 mN m^−1^, 26.2 ± 2.7 mN m^−1^, and 33.5 ± 5.3 mN m^−1^, respectively (*p* > 0.05). For DMPC, the MIP value for these three proteins was 29.6 ± 2.6 mN m^−1^, 29.8 ± 1.8 mN m^−1^, and 34.9 ± 3.7 mN m^−1^, respectively (*p* > 0.05) (Figure 6B). These results indicate that the deletion of the C-terminal peptide (aa 451–487) has no significant effect on the binding affinity or selectivity of (His)_6_-VpPLD.

However, significant differences were found between (His)_6_-VpPLD-Δ469-487 and (His)_6_-VpPLD-Δ434-487 for all phospholipid monolayers (Figure 6B). For DMPS, the MIP value for (His)_6_-VpPLD-Δ469-487 was 31.6 ± 3.4 mN m^−1^, while that for (His)_6_-VpPLD-Δ434-487 was 40.4 ± 4.6 mN m^−1^ (*p* < 0.05). For DMPG, the MIP value for (His)_6_-VpPLD-Δ469-487 was 30.1 ± 1.9 mN m^−1^, while that for the (His)_6_-VpPLD-Δ434-487 was 34.7 ± 2.1 mN m^−1^ (*p* < 0.05). For DMPE, the MIP value for (His)_6_-VpPLD-Δ469-487 was 26.2 ± 2.7 mN m^−1^, while that for (His)_6_-VpPLD-Δ434-487 was 45.1 ± 2.8 mN m^−1^ (*p* < 0.05). For DMPC, the MIP value for (His)_6_-VpPLD-Δ469-487 was 29.8 ± 1.8 mN m^−1^, while that for (His)_6_-VpPLD-Δ434-487 was 36.3 ± 3.5 mN m^−1^ (*p* < 0.05). The significant increase of the MIP values for all the phospholipid monolayers strongly indicates that the three-strand segment (aa 434–469) has a great negative effect on the interfacial binding to these phospholipid monolayers. The deletion of this peptide caused a much greater insertion of the protein into these phospholipid monolayers. In contrast, the single deletion of the helix (aa 469–487) had no significant effect on the interfacial binding properties.

Except for DMPG, there was no significant difference between (His)_6_-VpPLD-Δ451-487 and (His)_6_-VpPLD-Δ434-487 in the MIP for DMPS, DMPE, and DMPC. Also, no significant difference in the MIP for DMPS and DMPC was found between (His)_6_-VpPLD-Δ469-487 and (His)_6_-VpPLD-Δ451-487. This results further indicate that when the integrity of the three-strand structure was destroyed (deletion of amino acids 434–451 or 451–469), there was basically no effect on the binding affinity of (His)_6_-VpPLD to DMPS and DMPC.

Herein, the values of the synergy factor *a* for (His)_6_-VpPLD-WT and its C-terminal truncated mutants were all >0, indicating that a positive interaction occurred between the proteins and the substrates, despite the deletions (Figure 6C). Basically, the same trend as for the MIP was found for the synergy factor “*a*”. A statistical test was also performed to compare the synergy factor “*a*” of the different mutants for each phospholipid monolayers. As can be seen in Figure 6C, no significant difference could be seen between the synergy factor “*a*” of (His)_6_-VpPLD-WT and that of (His)_6_-VpPLD-Δ469-487 in the presence of DMPS and DMPC (Figure 6C), which was clearly supported by the MIP data (Figure 6B) (*p* < 0.05). In addition, the significant difference in the synergy factor “*a*” between (His)_6_-VpPLD-WT (0.29 ± 0.03) and (His)_6_-VpPLD-Δ469-487 (0.38 ± 0.03) in the presence of DMPG (*p* < 0.05) was also consistent with the results for MIP.

Considering the values of MIP for VpPLD-(His)_6_-WT and (His)_6_-VpPLD-WT on different phospholipids, for VpPLD-(His)_6_-WT, the highest MIP values were found for DMPS and DMPC, while for (His)_6_-VpPLD-WT, the highest values were found for DMPS and DMPE. However, no significant difference was found for DMPS, DMPC, and DMPE. For DMPC, a significantly higher value of MIP was found for VpPLD-(His)_6_-WT (37.4 ± 1.3 mN m^−1^) compared to the value obtained for (His)_6_-VpPLD-WT (29.6 ± 2.6 mN m^−1^) (*p* < 0.05). These results indicate that also the (His)_6_ tag has a great influence on the interfacial binding preference of VpPLD to different phospholipid monolayers. The effect of the (His)_6_ tag on the binding and hydrolytic activity of PLD was demonstrated in other studies [24,25,26]. The previous studies and the present results strongly indicate that the effect of the His tag should be taken into consideration when comparing the binding properties or the activities of proteins.

## 3. Materials and Methods

### 3.1. Materials

1,2-Dimyristoyl-*sn*-glycero-3-phosphocholine (DMPC), 1,2-Dimyristoyl-*sn*-glycero-3-phosphoethanolamine (DMPE), 1,2-Dimyristoyl-*sn*-glycero-3-phospho-*rac*-glyceol (DMPG), 1,2-Dimyristoyl-*sn*-glycero-3-phospho-l-serine (DMPS) were purchased from Larodon (Solna, Sweden). The expression vectors pET21a and pET28a were purchased from Stratagene (La Jolla, CA, USA). *Escherichia coli* Shuffle T7 Express Competent cells were purchased from New England BioLabs (Beijing, China). IPTG (isopropyl β-d-1-thiogalactopyranoside) and ampicillin were obtained from Sangon Biotech, Shanghai Co., Ltd. (Shanghai, China). Ni^2+^-NTA Sepharose fast-flow and anion exchange chromatography (Q-Sepharose XL) columns were purchased from GE Healthcare (Boston, MA, USA). Bicinchoninic acid (BCA) Protein Assay Kit was from Sangon Biotech, Shanghai Co., Ltd. (Shanghai, China). All other regents were of analytical grade.

### 3.2. Total Gene Synthesis of VpPLD and Construction of the Expression Plasmid

The target PLD protein sequence from VpPLD researched in the present study was deposited in the NCBI Protein database under the accession number EXJ48329.1. The deduced signal peptide was predicted by using SignalP 4.1 server [27]. The PLD gene encoding the mature peptide (with deletion of the first 18 amino acids (MLHTLSKFIFAFMFSVLS), named VpPLD-WT) was artificially synthesized according to the code usage of *E. coli* by Sangon Biotech, Inc. (Shanghai, China). The gene was ligated into the pET21a vector between the *Nde* I and *Xho* I restriction sites to yield the expression vector pET21a-VpPLD-WT-(His)_6_ (C-terminal 6× His tag). Meanwhile, the gene was also ligated into the pET28a vector between the *BamH* I and *Xho* I restriction sites to yield the expression vector pET28a-(His)_6_-VpPLD-WT (N-terminal 6× His tag). The constructed expression vectors were used to transform *E. coli* DH5α (Takara, Dalian, China). The recombinant plasmids were sequenced to ensure sequence accuracy and used to transform *E. coli* SHuffle T7. The molecular mass of the protein was predicted by using the Compute Mw tool at the ExPASy molecular biology web server of the Swiss Institute of Bioinformatics (http://www.expasy.org/) [28]. The predicted secondary structures of the N- and C-terminal peptides investigated in the present studies were obtained by using the I-TASSER server (http://zhanglab.ccmb.med.umich.edu/I-TASSER/) [29]. To ensure the accuracy of the predicted results, each sequence was analyzed using two additional online tools: Proteinprediction (http://www.predictprotein.org.) [30] and SSPro (http://scratch.proteomics.ics.uci.edu) [31].

### 3.3. Construction of Truncated Mutants of VpPLD

Truncation mutagenesis of the N-terminal and C-terminal of VpPLD was carried out by the overlap extension method with the constructed pET21a-VpPLD-WT-(His)_6_ plasmid or the pET28a-(His)_6_-VpPLD-WT plasmid as templates, respectively. Moreover, to ensure that the VpPLD and its mutants had no hydrolytic activities toward the phospholipid monolayers, a single-site mutation was further introduced in the key amino acid of VpPLD in its catalytic active site (H152A). The final products were digested with *Dpn*I and used to transform competent *E. coli* DH5α. The truncated mutants were sequenced to ensure their sequence accuracy. Then, the constructed plasmids were used to transform *E. coli* SHuffle T7.

### 3.4. Recombinant Protein Expression and Protein Purification

For the expression of recombinant wild-type VpPLD (VpPLD-WT) and its truncated mutants, *E. coli* SHuffle T7 cells harboring the corresponding constructed expression plasmids were grown at 37 °C in 1.0 L of Luria–Bertani (LB) medium containing ampicillin (0.05 mg/mL) and induced at an optical density of 0.8 at 600 nm by adding IPTG to a final concentration of 0.2 mM. After 20 h of induction at 20 °C, the cells were harvested, resuspended in 350 mL of 50 mM Tris-HCl (pH 8.0), and disrupted by sonication (Ultrasonic processor UH-950A, Tianjin Autoscience instrument Co., Ltd., Tianjin, China). The cell lysates were then centrifuged at 10,000 rpm for 20 min to remove the insoluble cell debris, and the supernatants were used for further purification.

For further purification of VpPLD-WT and its various mutants, the supernatants were filtered through 0.45 µm filters. Each supernatant was loaded onto Ni^2+^-NTA packed columns and washed with buffer A (50 mM Tris-HCl, 100 mM NaCl pH 8.0) containing 5 mM imidazole (final concentration). The target proteins were then eluted with buffer B (50 mM Tris-HCl, 100 mM NaCl, 200 mM imidazole pH 8.0). The fractions containing the proteins were then loaded onto Q-Sepharose XL columns and washed with buffer (50 mM Tris-HCl, 300 mM NaCl, pH 8.0). The target proteins were then eluted with the buffer (50 mM Tris-HCl, 500 mM NaCl, pH 8.0). The samples were analyzed by using 12% SDS-PAGE. Protein concentration was determined with the BCA Protein Assay Kit.

### 3.5. Binding of VpPLD-WT and Truncated Mutants to an Air–Water Interface in the Absence of a Phospholipid Monolayer

The measurements of the binding of VpPLD-WT and its various mutants to an air–water interface were performed using a Microtrough from Kibron (Helsinki, Finland). Microtrough containing 1.2 mL buffer (50 mM Tris, pH 8.0) in the subphase was used for the protein binding measurements. Different concentrations of proteins were injected into the subphase. A magnetic stirrer (diameter 0.5 cm) was used to stir the subphase at 100 rpm. The kinetics of protein binding to the air–water interface was monitored until the equilibrium surface pressure (Π_e_) was reached. Then, the values of maximum surface pressure increase (Π_max_) were recorded. When Π_max_ were plotted as a function of subphase protein concentration, the optimal protein concentrations used for the following MIP experiment could be obtained. In the presence of these protein concentrations, no increase in surface pressure was observed when the protein concentration was further increased, which indicated that surface saturation had been reached.

### 3.6. Binding of the VpPLD-WT and Its Truncated Mutants to Various Phospholipid Monolayers

In order to determine the effect of the head groups of the phosphatidyl moieties on the insertion of the proteins into phospholipid monolayers, four kinds of phospholipids with the same acyl chain in *sn*-1 and *sn*-2 positions were chosen (DMPS, DMPE, DMPC, DMPG). Various phospholipids solubilized in chloroform were slowly spread on the surface of a buffer (50 mM Tris, pH 8.0) poured in the wells of a multi-wells plate, until the desired surface pressure was reached. The time for solvent evaporation and for the film to reach equilibrium varied with the type of lipid, the spreading volume, the initial surface pressure, and the lipid concentration. Then, the proteins were injected into the subphase underneath the phospholipid monolayers at different initial surface pressures (Π_i_) until the equilibrium surface pressure (Π_e_) was reached. This allowed the calculation of the surface pressure increase (ΔΠ = Π_e_ − Π_i_). The plot of ΔΠ as a function of Π_i_ allowed determining the MIP and synergy factor *a* by extrapolating the regression of the curve to the x-axis. The values of MIP, synergy factor *a*, and each value’s uncertainty (calculated with a confidence interval of 95% from the covariance of the experimental data on the linear regression) were all determined by using a freely accessible web tool (http://www.crchudequebec.ulaval.ca/BindingParametersCalculator/).

## 4. Conclusions

The present results indicate that the N-terminal segment of PLD containing first 25 amino acids exerts a positive effect on the binding selectivity and affinity of VpPLD for DMPS and DMPC and a negative effect for DMPG. The C-terminal amino acid sequence (aa 434–469) exerts a negative effect on the interfacial binding to various phospholipid monolayers. Further study, using polarization modulation infrared reflection absorption spectroscopy (PM–IRRAS), will allow the determination of the secondary structure and of the orientation of the peptides in the presence of phospholipid monolayers. Moreover, a follow-up study, employing site-directed mutagenesis on the amino acids of the N- and C-terminal peptides, is necessary for the detailed functional analysis of this enzyme.

## Figures and Tables

**Table 1:** Analysis of the amino acid content of the N- and C-terminal sequences of PLD from *V. parahaemolyticus.*

Amino Acids Number	Amino Acid Sequence ^1^	Proportion of Amino Acids		
		Hydrophobic	Polar	Charged
1–25	**ACSSIESNQPSEKSTTFHFGYQQDS**	24% (6/25)	60% (15/25)	16% (4/25)
26–34	**VLAHYFEAY**	56% (5/9)	22% (2/9)	22% (2/9)
434–450	**SQLKDNAYRLTLRDGDI**	35% (6/17)	30% (5/17)	35% (6/17)
451–468	**VWTNTKTGEEYDSEPEAG**	33% (6/18)	33% (6/18)	33% (6/18)
469–487	**VFRRLGAWFSGILPIEDQL**	68% (13/19)	11% (2/19)	21% (4/19)

^1^ Hydrophobic amino acids are presented in red, polar amino acids in green, and charged amino acids in purple.
